# Assessment of Minimum Stable Areas for Young Ostriches According to Animal Welfare Legislation

**DOI:** 10.3390/ani15040582

**Published:** 2025-02-18

**Authors:** Sabrina Rückschloss, Robin N. Schüttpelz, Rüdiger Korbel

**Affiliations:** 1 Clinic for Birds, Small Mammals, Reptiles and Ornamental Fish, Ludwig-Maximilians-Universität München, 85764 Oberschleißheim, Germany; vorstandsassistenz@vogelklinik.vetmed.uni-muenchen.de; 2 Institute of Statistics, Ludwig-Maximilians-Universität München, 80539 München, Germany

**Keywords:** ostrich, barn area, animal welfare legislation, ostrich husbandry, ratites

## Abstract

Regarding the ongoing global development of commercial ostrich husbandry, it is necessary to establish science-based recommendations for the appropriate treatment and welfare of ostriches. In this study, we determined the minimum housing area required for ostriches aged between 6 and 15 months. The planimetry revealed that the animals in this age group cover an average area of 0.31 m^2^ to 0.57 m^2^ with their bodies. To investigate the effects of space differences, the animals were divided into three groups and kept in 2.5 m^2^, 5 m^2^, and 10 m^2^ of available space per animal. When comparing their weight and growth, a deficit was found in the animals that were provided with a housing area of 2.5 m^2^. Furthermore, the animals exhibited differences in their integument and plumage score. The ostriches with the smallest housing area showed poorer scores in the plumage and integument than the animals with more space. At the conclusion of this study, the animals were slaughtered, and their carcass weights were compared. Similarly, lower carcass weights of the animals with a 2.5 m^2^ barn area were observed. Prior studies have indicated that long-term stress leads to immune deficiencies associated with the inadequate development of the immune organs. However, a preliminary examination of the heart and spleen did not reveal any evidence for this. Further ethological studies are necessary to provide specific recommendations for minimum housing areas for ostriches.

## Simple Summary

The practice of ostrich farming has been a global phenomenon since 1860. However, in comparison to other poultry species, there is still a paucity of scientific knowledge regarding the welfare of ostriches. This became evident in 2019 during the revision of the German guideline for keeping ratites (“Gutachten über Mindestanforderungen an die Haltung von Straußen, Nandus, Emus und Kasuaren”). The current standards for the space requirements of ostriches are based on empirical observations. Accordingly, this study employed a planimetric approach to determine the area covered by ostriches aged 6–15 months (the ostrich fattening period). In order to evaluate the potential impact of differing stable areas on the health and productivity of ostriches, further comparative studies were conducted on three groups of ostriches kept in stalls measuring 2.5, 5, and 10 m^2^ per animal. The findings indicate that a barn area of 2.5 m^2^ per bird within the 6–15-month age range is associated with impaired growth and reduced performance in terms of body condition score parameters. Conversely, the data reveal minimal differences in growth and performance between birds housed in spaces measuring 5 m^2^ and 10 m^2^ per animal. Additionally, notable divergences were observed in the plumage and integument scoring between the group with a housing area of 2.5 m^2^ and the other groups.

## 1. Introduction

The ostrich industry has existed for around 160 years, with its beginnings in South Africa, and is therefore a relatively young agricultural sector worldwide. In the early days of ostrich farming, the focus was on feather production [1]; they were mainly used for jewelry and decoration. In 1863, the demand for ostrich feathers was such that a new branch of farming developed in South Africa. The First World War led to the collapse of the ostrich industry, which did not recover until the mid-1970s [2,3]. Ostrich products, especially ostrich leather and meat, are now marketed worldwide. The feathers are no longer among the main products. The global upswing has also led to the development of ostrich farming in other countries, including in Europe [3,4,5]. In 1980, ostrich husbandry boomed in Europe and became a significant agricultural branch [6]. The ostrich industry reached its absolute peak in the mid-1990s. However, after changes in fashion trends, the prices of ostrich leather, in particular, fell. Today, ostriches are kept on all five inhabited continents, mainly for meat production. Table 1 provides a list of the number of ostriches kept between 2000 and 2018/2019 [3], along with the main production countries worldwide. Due to the lack of official statistics, the numbers of ostriches kept and listed in Table 1 were gathered by the producers. However, one can see from these figures that ostrich farming has become more important in Europe. As a result of this upswing and the increase in ostrich farming, it is necessary to intensify the research in this field [3]. Compared with other livestock species, there is an immense need to catch up regarding animal welfare, feeding, housing, and rearing practices in global ostrich husbandry.

The stable space available is fundamental for animal welfare in ostrich husbandry. A sufficiently large barn space ensures that the ostriches visit the barn for protection from adverse weather conditions or other exceptional situations [7]. In addition, knowledge of the required housing area in ostrich husbandry is necessary, especially in connection with any official housing regulations, e.g., the legislation regarding avian influenza. The required minimum stable spaces according to the currently applicable legal provisions (the Council of Europe Recommendation on the Keeping of Ostriches (1979) and the Expert Opinion on Minimum Requirements for the Keeping of Ostriches, Nandus, Emus, and Cassowaries, Federal Ministry of Food and Agriculture (2019)) are shown in Table 2 [7,8]. Additionally, the recommendations of the German Veterinary Association for Animal Welfare (TVT) [9], the Federal Association of German Ostrich Breeders (BDS), and an expert group from the Faculty of Veterinary Medicine of the Ludwig Maximilian University of Munich (LMU) [10] are listed. The listed requirements of the Council of Europe correspond to knowledge that is around 20 years old and somewhat outdated [7]. In addition, both the requirements of the Council of Europe and the current husbandry guidelines are based on empirical values [7,8]. Scientific studies on the use of the barn areas and the resulting space requirements have not yet been practically executed. For this reason, more and more institutions in Germany (the TVT, BDS, and LMU) have been addressing the issue of minimum barn areas and publishing their own recommendations [9,10,11]. As demonstrated in Table 2, the required housing area for 6–15-month-old ostriches is between 2 and 10 m^2^/animal. Due to widely differing opinions, there is no uniform definition of minimum stall areas, resulting in discrepancies between livestock owners, veterinarians, and veterinary offices.

For this reason, we used planimetric measurements to determine the area required for 6–15-month-old ostriches based on their body size. In order to evaluate potential stress-induced physical effects on growth, plumage and skin condition, organs, and carcass quality caused by different housing areas, corresponding studies were carried out on ostriches kept in different housing areas.

## 2. Materials and Methods

### 2.1. Animals and Experimental Design

The present study was planned to specify the minimum barn area needed for ostriches aged between 6 and 15 months, interpreted in terms of animal welfare. This study was carried out in accordance with the German animal welfare regulations and with permission from the German authorities (reference number: ROB-55.2-2532.Vet_02-20-91).

The experimental animals were artificially brooded and hatched on an ostrich farm in Southern Germany, where this study was also conducted. The farm was established in 1993 and includes approximately 500 ostriches on an area of ~20 ha; it runs its own brooding, rearing, and slaughtering, certified according to Regulation (EC)853/2004 [12]. There were six groups of breeding animals from which the chicks used for this study were lineally descended. The chicks were reared in a group of 40 under the care of the same persons who maintained the animal experiment, in line with the legal requirements for ostrich farming in Germany [7]. All of the ostriches used for this investigation were clinically healthy. In order to minimize the stresses of handling during this study, the animals were accustomed to dealing with their handlers. Birds exhibiting high levels of fear or aggression towards humans were not selected for this project. Every person who stayed in contact with the experimental animals had a certificate of expertise in ostrich husbandry. To imitate economic ostrich husbandry, we chose only crossbreeds of blue-necked ostriches (*Struthio camelus australis*) and black-necked ostriches (*Struthio camelus domesticus*). In addition, we did not care about the sex of the experimental animals.

To satisfy the 3Rs principle (replacement, reduction, and refinement) and because of the high demand for pasture area, we kept the number of experimental animals as low as possible [13,14]. For this basic research, we split 24 ostriches into three groups of 8. A transponder was surgically implanted subcutaneously in the animals at an early age to facilitate clear identification. All groups were housed in the same barn in different departments. Every department was filled with straw and had an open exit to an adjoining pasture of 2500 m^2^. The three groups could hear and see one another. Because they were housed in an open stable, whose roof was covered with light panels, there was no need for additional artificial illumination to simulate the circadian rhythm. The constant access to a pasture ensured the fulfillment of the daily requirement for natural light. The climatic conditions in the barn corresponded to those of the environment. Thus, there were average temperatures of 2.6 °C in winter and 19.3 °C in summer, combined with an annual average relative humidity of 85% [14]. Water and food (a mixture of grass silage, maize silage, and grain meal) were provided ad libitum. The three departments only differed in the size of their barn area. In accordance with the recommendations of the BMEL, LMU, and the Council of Europe (Table 2), we investigated the effects of stocking densities of 2.5 m^2^/ostrich, 5 m^2^/ostrich, and 10 m^2^/ostrich on the ratites. This resulted in total barn areas of 20, 40, and 80 m^2^ for Groups 1, 2, and 3, respectively. The investigations of this animal experiment included planimetry, height and weight measurements, an assessment of injuries and plumage damage, and a final assessment of the carcass, heart, and spleen to determine the effects of high stocking density. The experimenters also recorded mortality, illnesses, and daily feed intake. After data collection, the results of the three groups were compared.

Due to increased evidence of avian influenza infections in wild birds near the ostrich farm during the time of the study, meaning an increased risk of infection to the experimental animals, we had to protect the ostriches from contact with wild birds at times. For this reason, the animals were kept in their barns for 48 days in spring. During this time, the exits to the pastures were hung with nets so that the ostriches still had enough daylight and the climate conditions of the environment were maintained. There were no additional changes to the size and equipment of the departments or in the feeding of the three groups.

### 2.2. Planimetry

**Figure 1:**
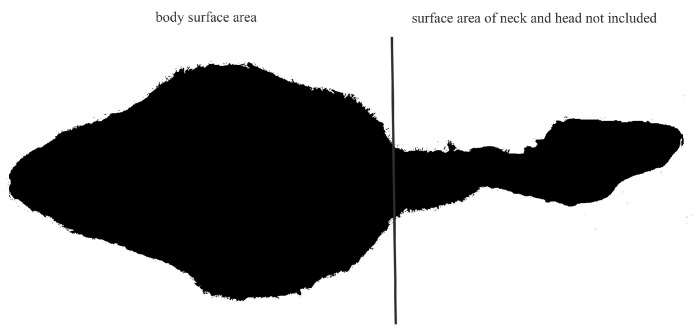
Representation of body parts used to calculate surface area.

The area of the ostriches occupied by their bodies was determined as a part of their weight and size measurements at three-month intervals. In order to minimize stress on the animals, the images were taken in the stabled area, which was familiar to them. To ensure that the results were not distorted by the animals’ feathering, the temperature during the photoshoots was maintained at a level that did not cause excessive fluffing. For the most accurate representation of the standing animals, all photographs were taken from a stationary position above the subjects (camera type: Lumix, DMC-FZ 100, Panasonic Corporation, Kadoma, Japan) [15]. The camera was positioned at a height of 2 m above the ground for the 6- and 9-month-old subjects, while the 12- and 15-month-old subjects were photographed from a height of 3 m above the ground. To facilitate the subsequent scaling of the images, a standard length of 20 cm was applied to each image. The photographic images were analyzed using the software ImageJ (Version 1.54j), which was employed to transform them into monochromatic images. Following this, the number of pixels of the imaged standard was determined for the subsequent area calculation. Upon modifying the threshold value of the image, the program was able to capture the body area of the animal in pixels. With the data collected from the standard, the area covered could then be calculated from the number of pixels in the animal’s body. The surface area of the entire body of the animal was measured, beginning at the level of the neck (Figure 1).

### 2.3. Weight

The 24 experimental animals were weighed four times at intervals of three months between November 2021 and September 2022. The eight ostriches of each group were caught and blinded with a hood over their heads. This clinically proven and professionally established technique made their handling easier. After blinding, two persons led the ratites backwards onto the Marsden scales used for large animals up to 150 kg (model V110, Marsden Weighing Machine Group Limited, Niebüll, Germany). For weighing, the ostriches had to stand still for approximately two seconds until their weight was shown on the display.

### 2.4. Scoring

Skin damage and plumage quality can be professionally assessed using a scoring system [16]. Each influences the other, and both are important indicators of animal welfare. On the one hand, the wellbeing of the animals decreases during an illness; on the other hand, with a lack of wellbeing, diseases are more likely to occur because of immunosuppression [17]. Especially in poultry flocks, deficiencies in husbandry conditions and diseases can be detected at an early stage by assessing plumage quality and injuries. In this study, we used this method to evaluate the effects of the different barn areas on the wellbeing of the ostriches. In order to circumvent the potential for disparate evaluations, the comprehensive evaluation of the integument and plumage was conducted by a single individual.

#### 2.4.1. Plumage Quality

The plumage was scored when the ostriches were caught for weighing. For this purpose, the blinded ratites were placed in front of a bale of straw and slightly fixed by one person. The investigation was carried out by a second person. For an objective and comparable evaluation, we used a modified scoring method provided by Aerni [18,19]. This method comprises 3 body parts for plumage condition (breast, back, and wings) and a scoring system with a rating of 1 (perfect plumage), 2 (isolated feathers damaged or missing; no denuded area), 3 (numerous feathers damaged or missing; denuded area up to 5 × 5 cm), or 4 (damaged plumage; denuded area greater than 5 × 5 cm). A total feather damage score was calculated for each animal by adding the 3 scores together (range: 3 to 12). To compare the general plumage condition between the 3 groups, a group score was calculated by adding the 8 individual animal scores (range: 24–72).

#### 2.4.2. Injuries

The procedure for scoring skin damage was the same as for plumage condition; however, for the scoring of injuries, we did not distinguish between body parts. We only scored the whole body, with a score of 1 (no injuries), 2 (maximum 2 injuries up to 5 × 5 cm), or 3 (more than 3 injuries up to 5 × 5 cm; minimum 1 injury greater than 5 × 5 cm). Similarly, a group score was calculated by adding the individual animal scores together, facilitating comparison between the groups [20].

The total group score for each group was determined by adding the scores for injuries and plumage. The resulting total group scores ranged from 32 to 120.

### 2.5. Carcass Inspection

#### 2.5.1. Slaughtering

Before slaughtering, the animals were reviewed by an official veterinarian according to Regulation (EC) 1099/2009 [21] in order to identify any abnormal conditions. All 24 experimental ostriches were killed at the end of this study, at an age of 15 months. For this purpose, the animals were led to the company’s own slaughterhouse that was located on the farm. Each ratite was stunned using electric tongs, which were applied vertically on their heads. The stunning current flow lasted for four seconds, with a current of 500 mA. To ensure a better flow of electricity, the birds were blinded with a cloth hood soaked in isotonic saline solution. Immediately afterward, the hanging and bleeding took place within 20 s. The ostriches were hoisted onto an overhead trail, and a high neck cut was performed, which opened both carotid arteries and both jugular veins, followed by a stab to the heart. After death, the feathers were removed via pulling. To minimize microbial contamination, the head, feet, tail, and hide were also removed [22,23,24]. The carcasses were moved into a separate evisceration room, where the thoracic contents and viscera were removed, and the carcasses were weighed.

#### 2.5.2. Carcass Weight

The carcass was weighed after removing the feathers, head, feet, tail, hide, and viscera; it was pushed to the part of the overhead trail where the scales are integrated. The weight of the carcass was shown on a digital display, with an accuracy of 100 g.

#### 2.5.3. Inspection of Heart and Spleen

Directly after slaughter, we examined the weight and volume of the heart and spleen as indicators of an increased stress load triggered by an inadequate stable area.

First, we determined the weight with a calibrated Bizerba scale (type: EW 100E, Bizerba SE & Co. KG, Balingen, Germany). The length and width of both organs were then measured with a ruler at the most extended points. After this, the displacement method was used to determine the volume of the organs as accurately as possible. We used a five-liter measuring jug and filled it with two liters of water. After the organ was placed in the measuring beaker and completely covered with water, the volume was read off the scale. The volume of the organs was then calculated by subtracting the starting volume of two liters from the final volume.

### 2.6. Statistical Analyses

The values determined in this study were analyzed in cooperation with the LMU’s statistical consulting laboratory (StabLab LMU) and transferred into graphical representations. The data comparison between the three groups was primarily carried out by comparing the calculated mean values and their standard deviations. We used the linear regression model to calculate the covered areas as a function of weight.

## 3. Results

Throughout the course of this study, the avian subjects were observed daily for any indications of disease. During this period, the 24 experimental ostriches did not exhibit any signs of illness, and no mortalities occurred.

The consumption of feed by the three groups in the enclosure is illustrated in Table 3.

The findings of the investigation revealed no substantial discrepancies between the three groups. During the period in which the animals were housed for the purpose of safeguarding against avian influenza, a significant increase in food intake was observed in all three groups.

### 3.1. Planimetry

The results of the planimetric investigation, listed in Table 4, demonstrate that the surface area covered by the animals’ bodies exhibited slight variations between the groups. The mean body surface area of the ostriches in Group 1 was 0.28 m^2^ at six months of age, while that of the ostriches in Groups 2 and 3 was 0.31 and 0.33 m^2^, respectively. Within Group 1, an increase in coverage was observed: the surface area increased from 0.34 m^2^ at 9 months of age to 0.45 m^2^ at 12 months of age and subsequently to 0.56 m^2^ at 15 months of age. Groups 2 and 3 exhibited a similar pattern of development in terms of surface coverage: at 9 months of age, the observed values were 0.37/0.38 m^2^; at 12 months they were 0.49/0.50 m^2^; and at 15 months, they were 0.55/0.61 m^2^, respectively.

A planimetric examination of the 24 six-month-old ostriches revealed an average body coverage area of 0.31 m^2^. As the animals matured, this area exhibited a distinct growth pattern: 9 months—0.36 m^2^, 12 months—0.48 m^2^, and 15 months—0.57 m^2^. The standard deviation of all measurements was low, at 0.05–0.09 m^2^.

### 3.2. Weight

**Figure 2:**
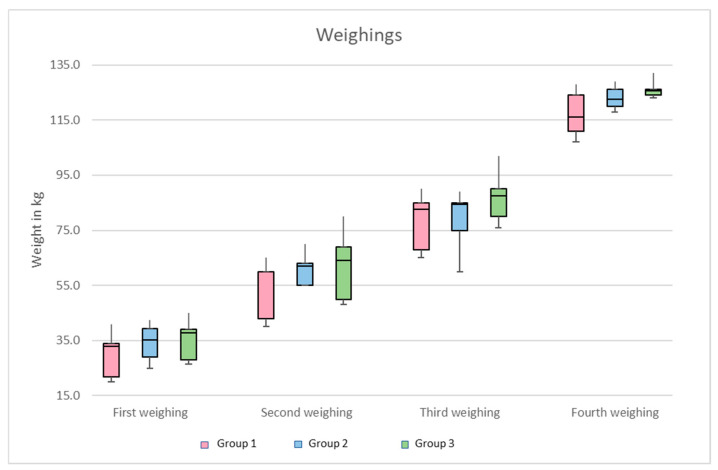
Comparison of weight development in Groups 1, 2, and 3.

When assembling the three groups, care was taken to ensure that the weights of the individual animals were similar. However, due to the limited number of ostriches available and the different genetic materials of different breeding animals, the weights of the individual animals ranged from 20 to 45 kg at the beginning of the study. The mean initial body weight of the subjects in Group 1 was 30.4 kg at six months of age, while in Groups 2 and 3, it was 35.0 and 35.8 kg, respectively (Figure 2). The lower average weight of Group 1 was also evident during the following three weighings at three-month intervals (54 kg; 79.6 kg; 117.5 kg body mass), while the weights of Groups 2 and 3 were similar (61.8 kg/63.1 kg; 88.6 kg/87.5 kg; 123.3 kg/126.1 kg body mass) (Figure 2). However, the development of the weight differences from the lightest to the heaviest animals within a group was striking. The measurement at the beginning of this study showed a maximum weight difference of 21 kg within Group 1, 17.6 kg within Group 2, and 18.5 kg in Group 3. These weight differences increased significantly during the second and third measurements, especially in Groups 2 (15 kg; 39 kg) and 3 (32 kg; 26 kg), while they remained relatively constant in Group 1, with a weight difference of 25 kg. Despite that, the last weighing of the 15-month-old ostriches still showed a weight difference of 21 kg in Group 1, while in Groups 2 and 3, the measured weight differences were only 11 kg and 9 kg, respectively (Figure 2).

**Figure 3:**
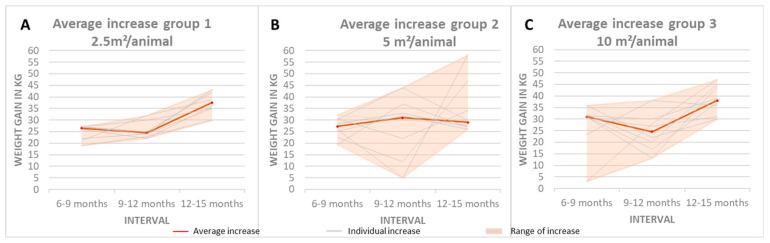
Weight gains at 6–9 months of age, 9–12 months of age, and 12–15 months of age for (**A**) Group 1, (**B**) Group 2, and (**C**) Group 3.

A further calculation was performed to determine the weight gain over the course of the three-month period. The mean weight gains observed during the three measurement intervals in Group 1 exhibited a nearly constant range between the highest and lowest gains. In Groups 2 and 3, the discrepancy between the highest and lowest rates of increase was more pronounced due to the influence of outliers. It is evident that the rate of increase observed in Group 1 (24.0 kg) during the initial interval was markedly inferior to that observed in Groups 2 (27.3 kg) and 3 (31.0 kg). The highest average weight gain for Groups 1 (37.5 kg) and 3 (38.0 kg) was exhibited by animals between the ages of 12 and 15 months, while Group 2 exhibited the highest average weight gain (31.0 kg) between the ages of 9 and 12 months (Figure 3A–C). Throughout the observation period, Group 1 experienced the lowest average weight gain of 87.1 kg, while Group 3 demonstrated the highest average weight gain of 90.3 kg; Group 2 showed an average weight gain of 88.3 kg over the entire observation period.

**Figure 4:**
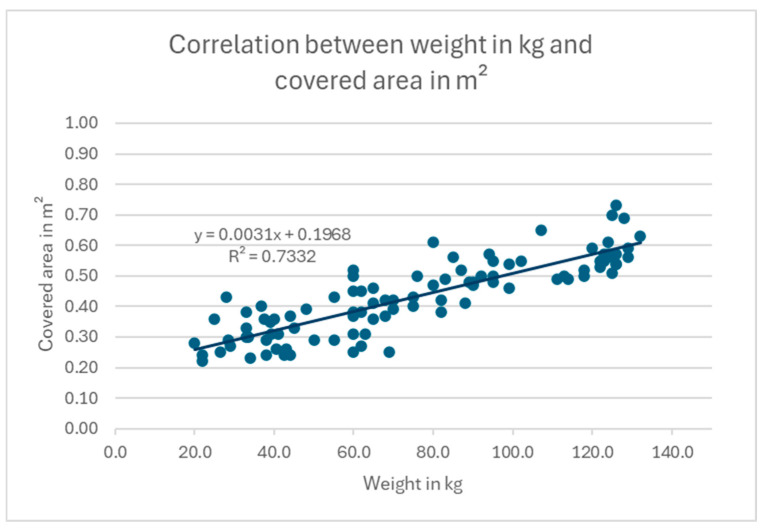
Mean surface area (m^2^) covered by ostriches aged between 6 and 15 months.

Following the completion of the planimetric investigation and the weight measurement, an attempt was made to establish a correlation between the values determined. In this age range, a nearly linear relationship was identified between live weight and the area covered by the animal’s body in the standing position (linear regression with a correlation coefficient of R^2^ = 0.73) (Figure 4).

### 3.3. Scoring

None of the three groups showed any major injuries or plumage damage during our experiment.

The first examination of the six-month-old ostriches from all three groups showed no injuries or plumage damage, and this remained the case after the first six months of the study; no injuries were found either. However, after another three months, three birds (37.5%) in Group 1 were found with isolated broken feathers in the chest area; their plumage score was therefore assessed as two. These damaged areas were not able to regenerate by the time of the last examination. In addition, during the last scoring of the 15-month-old ostriches, an animal with a minor injury, rated two, was found in Group 1. This injury was surrounded by a featherless area measuring approximately 10 cm × 7 cm, rated three (Table 5).

Three animals with minor plumage damage were identified in the second and third investigations. In the fourth investigation, one of the previously injured animals exhibited a deterioration in plumage condition and a minor injury.

In the second group, no birds with feather damage or injuries were observed throughout the entire observation period (Table 6).

In the third group, an animal with a small scratch (1 cm × 3 cm) on the right femur, rated two, was also found during the last scoring session. We did not find any evidence of additional feather damage or injuries in Group 3 (Table 7).

### 3.4. Carcass Inspection

#### 3.4.1. Carcass Weight

**Figure 5:**
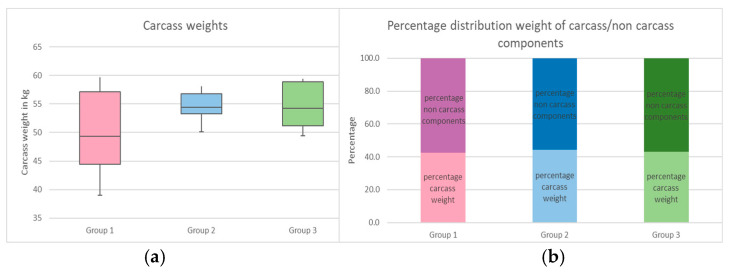
(**a**) Distribution of carcass weight. (**b**) Percentage distribution of carcass share: Group 1 (2.5 m^2^/animal), Group 2 (5 m^2^/animal), and Group 3 (10 m^2^/animal).

The disparate weight developments observed in the living animals across the three groups can ultimately be elucidated through a comparison of the carcass weights. Figure 5 illustrates a markedly greater dispersion of the values recorded in Group 1 compared to Groups 2 and 3. The mean slaughter weight of Group 1 was 50.1 kg, which was considerably less than that of Groups 2 (54.5 kg) and 3 (54.3 kg). The average percentage distribution of usable carcass parts and non-usable components showed little difference between the individual groups (Figure 5). The results of the investigation demonstrated that Groups 1–3 exhibited body yields of 42.4%, 44.2%, and 43.0%, respectively.

#### 3.4.2. Inspection of the Heart and the Spleen

No statistically meaningful differences were observed in the measurements of the heart and spleen across the three groups.

The absolute weights of the hearts of animals from Groups 1 and 2 were similar, with a mean of 850.1 g and 867.8 g, respectively. In contrast, the animals in Group 3 had a mean heart weight of 937.0 g. A comparison of the heart weights with the live weights in percentage terms showed that all the animals had similar values. The figures for Groups 1–3 were 0.72%, 0.71%, and 0.74%.

A comparable effect can be observed with regard to the weights of the spleens. With consideration to the absolute weights, discrepancies between Groups 1, 2, and 3 were identified (38.25 g, 35.38 g, and 37.13 g, respectively). A calculation based on the animals’ live weight indicated only a slight difference in the spleen weights (Table 8).

The results of the volume measurements also demonstrated minimal discrepancies between all three groups. The heart volumes of the groups were 1338 mL (Group 1), 1368 mL (Group 2), and 1335 mL (Group 3). A comparable trend was observed in the spleen volumes, which were 30.0 mL (Groups 1 and 2) and 30.6 mL (Group 3).

## 4. Discussion

The German animal welfare legislation and other different recommendations have so far only been based on practical experience and habits, rather than scientifically sound knowledge. In order to establish minimum stable space requirements for ostrich husbandry, it is essential to have a clear understanding of the surface area required by the animals’ bodies [15,25]. The present study provides an overview of the requisite stable space available and the possible consequences of inadequate stall space. To this end, a variety of investigations were conducted.

The planimetric examination of the 24 experimental subjects revealed that, during the fattening period between 6 and 15 months, a linear direct correlation exists between weight gain and the surface area covered by the ostriches. The data obtained from this investigation demonstrated that a 6-month-old ostrich, with an average body weight of 33.7 kg, occupied an area of 0.31 m^2^. With average live weights of 59.6 kg and 85.3 kg, 9- and 12-month-old ostriches require average areas of 0.36 m^2^ and 0.48 m^2^, respectively. By the end of the study, the 15-month-old ostriches exhibited an average live weight of 122.3 kg, covering an area of 0.57 m^2^. Over the course of nine months, the live weight of the animals quadrupled, while their surface coverage nearly doubled. This growth trajectory can be attributed to the animals’ rapid height increase during this developmental stage. Our investigation yielded an average increase in shoulder height of 40 cm. A comparable growth trajectory was observed by N.C. Meyer [26] in a study on the development of a body condition score for ostriches; the author determined that the mean increase in shoulder height for ostriches between the ages of 6 and 15 months was 39.25 cm, while the mean increase in weight was 70.1 kg [26]. These data form the basis for setting a minimum stall size for young ostriches. In a study by Hughes [27], the space requirements of laying hens were investigated, and the additional space needed for space-consuming behaviors such as turning and stretching was determined. It was also determined that individual spacing between animals should be possible. Planimetric studies on turkeys showed that, with existing stocking density regulations, the animals have approximately twice as much floor space available as they cover with their bodies [25]. If this regulation is also applied to ostriches, the present study shows a minimum space requirement of 0.62 m^2^ for 6-month-old ostriches, 0.72 m^2^ for 9-month-old ostriches, 0.96 m^2^/animal at the age of 12 months, and 1.14 m^2^/animal for 15-month-old ostriches. This corresponds to a stocking density of 1.6 six-month-old ostriches/m^2^, 1.3 nine-month-old ostriches/m^2^, 1.0 twelve-month-old ostriches/m^2^, and 0.9 fifteen-month-old ostriches/m^2^. However, when determining the amount of space required for an ostrich, it is important to remember that even hand-raised ostriches still have a strong wild animal character and, therefore, are more prone to flight, panic, and defensive reactions [10,28]. In conjunction with inadequate dimensions of the stables, this can result in a deterioration of the overall condition of the animals due to prolonged stress. Accordingly, this study endeavored to ascertain the clinical health status of ostriches held on different pasturelands, in conjunction with their individual development trajectories, through the determination of supplementary parameters.

As postulated by Troxler [29], there are enduring reciprocal interactions between animals and their environment, which are reflected in the morphology, physiological response, and behavior of the animals. An inappropriate environment results in behavioral disorders that manifest to varying degrees. In a study of behavioral disorders in Canada, Samson [30] observed that feather picking in ostriches is caused by overcrowding or boredom due to inadequate foraging opportunities. In the context of the present investigation, the plumage and potential injuries sustained by the animals during the growth phase period, which spanned a period of 6 to 15 months, were initially evaluated. The three groups of eight ostriches, occupying 2.5 m^2^, 5 m^2^, and 10 m^2^ per bird, exhibited no significant damage to their plumage or evidence of injury. One animal in Group 3 (10 m^2^/animal) exhibited a small abrasion on the right hind limb. This could be attributed to contact with the stall fixtures or the fencing. In light of the absence of further injuries or damage to the plumage, this observation can be considered inconsequential. However, after the three-month observation period, an increased number of birds with broken feathers in the chest area was noted in Group 1 (2.5 m^2^/bird). In one instance, a 10 cm × 7 cm area of bare skin developed in this region, accompanied by a minor skin injury. The loss of feathers in the chest does not indicate extensive feather pecking, since according to Samson [30], the affected areas are mainly around the back and tail. Rather, the cause of the feather loss is found in the design of the stable. The feeding places of the stallions are designed in the form of a feeding grid. When feeding, the animals lean their chests slightly against the slats of the fence. The feeding area was the same for all three groups, except that Groups 2 and 3 had significantly more space in this area. It is possible that, because of the reduced space available, the animals of Group 1 may have crowded against the fence when feeding, causing the aforementioned feather damage.

Other studies of the effects of high stocking densities on the behavior and welfare of poultry have shown reduced performance due to decreased feed intake and activity. Bessei published a study in 1999 entitled “Behaviour of broilers in relation to group size and stocking density” [31]; he also found that high stocking densities resulted in reduced feed intake and activity. Later, Bessei [32] noted that a smaller space or higher stocking density increases the temperature at the level of the animals and, thus, reduces their activity. To evaluate that factor in this study of ostriches, weight gain and daily feed intake were measured over the duration of the experiment. As shown in Table 3, there were no substantial differences in feed intake between the three groups. This result is consistent with the findings by Thomas et al. and Revindran et al. [33,34], who found no impairment of feed intake due to different stocking densities in their studies on broilers. Contrary to the findings of the present study, Shannawany, Dozier et al., and Tong et al. (2012) were able to demonstrate reduced feed intake when the stocking densities were increased [35,36,37]. However, it should be noted that each group in this study had a pasture area of 2500 m^2^ at its disposal. The natural behavior of ostriches includes foraging on the pasture, which takes approximately six to eight hours throughout the day [38]. This additional feed intake was not taken into account in the present study.

When the initial weights were determined, it was found that there were relatively large differences due to the different parental animals and, thus, different genetics. The animals of Group 1 weighed an average of 30.4 kg at the beginning of the experiment, while those of Groups 2 and 3 were 35.0 kg and 35.8 kg, respectively. Due to the significantly lower average weight of Group 1, it was difficult to compare the absolute weights in the further course of this study. Therefore, in this investigation, it was more meaningful to evaluate the development of the weight differences. While the weight difference between the lightest and the heaviest animals in Group 1 remained relatively constant between 21 and 25 kg throughout the experiment, Groups 2 and 3 showed small variations of 17.6 kg and 18.5 kg, respectively, at the beginning of the experiment. At the end of the study, both groups had a very small variation in weight, with maximum variations of 11 kg in Group 2 and 9 kg in Group 3. In addition, if we look at the average weight gain of all animals in a group, we can see that Group 1 had the lowest weight gain, at 87.1 kg, while Group 2 had a weight gain of 88.3 kg, and Group 3 had the highest, at 90.3 kg. These results show that the animals with the smallest barn area had a deficit in weight development compared to the other groups. While the weights of Groups 2 and 3 converged towards the end of the growth phase at 15 months, the relatively large variation in Group 1 remained. It is noteworthy that even animals in Groups 2 and 3 with low initial weights showed high gains during the experiment. In Group 1, however, the animals with low initial weights remained at a low weight level until the end of the experiment. In addition, the animal with the lowest weight gain in Group 1 was already conspicuous for feather damage and skin lesions. This suggests that the weaker animals in Group 1 were permanently suppressed by the stronger ones.

This assumption is corroborated by the findings of the carcass assessment. As illustrated in Figure 3, a markedly wider range of carcass weights was observed in Group 1 in comparison to the other two groups. While the discrepancy between the lowest and highest carcass weights in Group 1 was 20.7 kg, in Groups 2 and 3, it was only 8 kg and 10 kg, respectively. A comparison of the average utilization of slaughter animals reveals a deficit in the Group 1 dataset, where the mean value for the proportion of live weight converted to slaughter weight is 42.4%; in Groups 2 and 3, the corresponding figures are 44.2% and 43.0%, respectively. By taking into account the average slaughter rate and the average live weight, it is possible to calculate the absolute weight of the portion of the animal that can be put to use. This calculation yielded values of 49.82 kg for Group 1, 54.50 kg for Group 2, and 54.22 kg for Group 3. It can thus be inferred that the slaughter yield and, consequently, the performance of the ostriches in Groups 2 and 3 were significantly higher than in Group 1. The studies conducted by Morris et al. [24] and Polowska et al. [39] on the carcass yield of ostriches yielded partially higher values. The analysis of 14 animals aged 10–14 months and with an average live weight of 95.5 kg yielded a mean slaughter weight of 54.57 kg and a slaughter yield of 58.6% in the study conducted by Morris et al. [24]. The investigations conducted by Polowska et al. [39] yielded comparable results, with a slaughter yield of 49.0% and an average carcass weight of 47.6 kg at a live weight of 99.7 kg. In a study conducted by Bessei [31], a decline in slaughter yield was observed with increasing stocking density, although the reduction was relatively minor. Nevertheless, the author concluded that “the stocking density is the key issue for economical result of broiler production” [40]. In the present study, the stocking density may have been part of why the weight gains—and, ultimately, the slaughter weights—of Group 1 were significantly lower than those of Groups 2 and 3.

In order to determine the potential effects of different barn areas on the development of the heart and the spleen, the size and weight of these organs were recorded during the slaughter examination, and the percentage contribution of these organs to the live weight was calculated for better comparability, independent of the weight of the animals. Pope asserts that the measurement of the spleen’s weight in relation to the body weight is a conventional method for evaluating the immune status, which is influenced by chronic stress [41]. According to this, chronic stress has been demonstrated to result in a decline in immunocompetence, which is concomitant with a decline in spleen weight. The investigations conducted by Tong et al. and Heckart et al. yielded equivocal results on this matter. A modest tendency towards a decrease in spleen weight in response to high population density was discerned [37,42]. The present study was unable to demonstrate any substantial effects of varying population density on spleen weight. The percentage contribution of the spleen to the body weight after slaughtering was 0.03% in all three groups. These results are similar to those of Engku Azahan and Noraziah in 2001 [34], who reported values of 0.04% for the spleen and 0.9% for the heart. In this study, the proportions of the heart were 0.81%, 0.82%, and 0.89% in Groups 1, 2, and 3, respectively; the minimal differences in these proportions were not significant. In contrast, Aengwanich et al. were able to demonstrate an enlargement of the heart in broilers under the influence of chronic stress. In particular, an enlargement of the right ventricle and the right atrium due to an increased heart rate and increased pulmonary pressure was observed [43]. The findings of the present study suggest that the distinct stable spaces may not have exerted significant effects on the weights of the examined organs, a phenomenon that could be attributed to the extensive grazing area.

## 5. Conclusions

The objective of the present study was to obtain empirical data regarding the space requirements of ostriches within the age range of 6 to 15 months. The planimetric investigation served as the foundation for the evaluation of the minimum stable spaces required for young ostriches. The objective of subsequent investigations within this study was to ascertain the potential impacts of the varying spaces available. It was observed that the animals with the smallest stable space exhibited distinctive characteristics in both the qualitative assessment of plumage and the diagnosis of integument injuries. The preliminary examination of the heart and spleen did not reveal any unusual findings. The results of the present study suggest that a floor area of 2.5 m^2^ per bird in the 6–15-month age range is associated with impaired growth and reduced performance. In contrast, the data indicate minimal differences in growth and performance between birds housed in spaces of 5 m^2^ and 10 m^2^ per animal. However, it should be noted that, throughout the duration of this study, all animals had access to a pasture area of 2000 m^2^ per group. This suggests that the effects of the varying stable spaces may have been offset by the accessible pasture space. To draw well-founded conclusions, further targeted ethological investigations will be necessary.

## Figures and Tables

**Table 1:** Main ostrich-producing countries. Numbers are provided by producers; official statistics are mostly not available.

Non-European Countries				European Countries			
Year	2018/2019	2010	2000	Year	2018/2019	2010	2000
China	500,000 *	500,000 *	250,000 *	Ukraine	50,000 ****	1500 *	0
Brazil	250,000 *	450,000 *	0	Romania	10,000	1000	0
South Africa	130,000 **	250,000	300,000	Poland	3000	5000	0
Pakistan	100,000 *	0	0	Germany	2500	1750	1000
Iran	4000 *	0	0	Portugal	2000	2000	2000
Arabian countries/Emirates	25,000 *	0	0	Hungary	1500	1000	0
Botswana	15,000	0	0	France	1500	1500	500
New Zealand	15,000	15,000	10,000	Austria	1000	1000	500
Australia	15,000 ***	15,000 ***	30,000	Bulgaria	1000	0	0
Israel	0	1000	25,000	Italy	1000	2000	5000
Namibia	0	2000	25,000	Spain	1000	1500	7000
Zimbabwe	0	5000	55,000				

* For home regional market only; ** temporarily banned from export; *** exported to the USA and Japan only; **** not yet approved for the EU.

**Table 2:** Actual minimum barn area requirements of various European institutions [7,8,9,10,11].

	Federal Ministry of Food and Agriculture		TVT ^1^		Council of Europe		BDS ^2^		LMU ^3^	
Age	m^2^/bird	Minimum Floor Space (m^2^)	m^2^/bird	Minimum Floor Space (m^2^)	m^2^/bird	Minimum Floor Space (m^2^)	m^2^/bird	Minimum Floor Space (m^2^)	m^2^/bird	Minimum Floor Space (m^2^)
0–1 week			0.25	Not specified	0.25	5	0.25	Not specified	0.25	1
Up to 3 weeks					0.25–1.2	25				
Up to 8 weeks							0.25–1	5	0.25	1
Up to 12 weeks			0.25–1	Not specified					0.25–1	5
Up to 4 months	1.7	25								
Up to 6 months			2	Not specified	2–10	30	2	15	1–2	30
From 6 months	2.5	25	2–4	Not specified						
Up 12 months	2.5	25			10	30				
Up 14 months							4	15		
Up 16 months									3–5	30
Breeding birds	5	25	5	Not specified						
From 13 months					10	30				
From 15 months							5	15		
From 17 months									5	30

^1^ German Veterinary Association for Animal Welfare; ^2^ Federal Association of German Ostrich Breeders; ^3^ Faculty of Veterinary Medicine of the Ludwig Maximilian University of Munich.

**Table 3:** Daily feed intake of the mixture of grass silage, maize silage, and grain meal in the barn.

Age (Months)	Feed Intake (kg)		
	Group 1	Group 2	Group 3
6–7	8.32 (±0.83)	7.15 (±1.26)	9.79 (±1.22)
7–8	10.83 (±2.13)	11.28 (±3.45)	10.99 (±3.22)
8–9 ^1^	23.59 (±1.45)	22.31 (±2.41)	24.52 (±1.04)
9–10 ^1^	39.27 (±0.77)	41.88 (±0.82)	38.21 (±0.95)
10–11	38.98 (±1.33)	39.59 (±0.97)	38.52 (±0.73)
11–12	42.11 (±1.27)	43.14 (±0.83)	40.99 (±0.75)
12–13	45.21 (±0.74)	42.98 (±0.44)	44.84 (±0.53)
13–14	45.66 (±0.93)	43.12 (±0.56)	45.28 (±0.85)
14–15	46.33 (±0.75)	45.21 (±0.47)	47.73 (±0.56)

^1^ The ostriches were confined during the specified periods to mitigate the risk of avian influenza.

**Table 4:** Mean surface area coverage in square meters per animal at six, nine, twelve, and fifteen months of age.

Area Coverage, m^2^ (Mean Value ± Standard Deviation)				
	6 Months	9 Months	12 Months	15 Months
Group 1	0.28 (±0.05)	0.34 (±0.09)	0.45 (±0.06)	0.56 (±0.08)
Group 2	0.31 (±0.05)	0.37 (±0.07)	0.49 (±0.05)	0.55 (±0.07)
Group 3	0.33 (±0.06)	0.38 (±0.09)	0.50 (±0.07)	0.61 (±0.05)
Overall average	0.31 (±0.06)	0.36 (±0.09)	0.48 (±0.07)	0.57 (±0.07)

**Table 5:** Plumage evaluation and injury investigation of Group 1.

Group 1 (2.5 m^2^/animal)	Rating 2 (After 3 Months)	Rating 3 (After 6 Months)	Rating 4 (After 9 Months)
Plumage			
Breast	11	11	12
Back	8	8	8
Wings	8	8	8
**Total score of plumage**	**27**	**27**	**28**
Injuries	8	8	9
**Total group score**	**35**	**35**	**37**
Number of animals affected	3	3	3

**Table 6:** Plumage evaluation and injury investigation of Group 2.

Group 2 (5 m^2^/animal)	Rating 2 (After 3 Months)	Rating 3 (After 6 Months)	Rating 4 (After 9 Months)
Plumage			
Breast	8	8	8
Back	8	8	8
Wings	8	8	8
**Total score of plumage**	**24**	**24**	**24**
Injuries	8	8	8
**Total group score**	**32**	**32**	**32**
Number of animals affected	0	0	0

No evidence of feather damage or injury was observed.

**Table 7:** Plumage evaluation and injury investigation of Group 3.

Group 3 (10 m^2^/animal)	Rating 2 (After 3 Months)	Rating 3 (After 6 Months)	Rating 4 (After 9 Months)
Plumage			
Breast	8	8	8
Back	8	8	8
Wings	8	8	8
**Total score of plumage**	**24**	**24**	**24**
Injuries	8	8	9
**Total group score**	**32**	**32**	**33**
Number of animals affected	0	0	1

No injuries or feather damage was identified in the second and third examinations; however, in the fourth examination, a minor injury was diagnosed in one animal.

**Table 8:** Comparison of the average live weight, carcass weight, heart weight, and spleen weight of the animals of Groups 1 (2.5 m^2^/bird), 2 (5 m^2^/bird), and 3 (10 m^2^/bird).

Group Parameters	Group 1	Group 2	Group 3
Live weight (in kg)	117.5 ± 7.5	123.3 ± 3.6	126.1 ± 3.0
Carcass weight (in kg)	50.1 ± 7.2	54.5 ± 2.5	54.3 ± 3.6
Carcass %	42.4 ± 3.5	45.4 ± 3.9	43.1 ± 1.8
Heart weight (in g)	958.25 ± 144.82	1006.13 ± 116.13	1115.87 ± 120.35
Heart %	0.81 ± 0.09	0.82 ± 0.10	0.89 ± 0.10
Spleen weight (in g)	38.25 ± 7.07	35.38 ± 7.63	37.13 ± 10.52
Spleen %	0.03 ± 0.0065	0.03 ± 0.0066	0.03 ± 0.0085

## Data Availability

Data are contained within the article and Supplementary Materials.

## Supplementary Materials

The following supporting information can be downloaded at https://www.mdpi.com/article/10.3390/ani15040582/s1: Figure S1. Aerial view of the ostrich farm. Figure S2. Aerial view of the pasture area. Figure S3. Barn and animals of Group 1 at the beginning of the study. Figure S4. Barn and animals of Group 2 at the beginning of the study. Figure S5. Barn and animals of Group 3 at the beginning of the study. Figure S6. Barn and animals of Group 1 at the end of the study. Figure S7. Barn and animals of Group 2 at the end of the study. Figure S8. Barn and animals of Group 3 at the end of the study. Figure S9. Group 1, bird with injury. Figure S10. Group 1, bird with loss of feathers. Figure S11. Group 3, bird with injury. Table S1. Carcass weights. Table S2. Group 1, weights and gain. Table S3. Group 2, weights and gain. Table S4. Group 3, weights and gain. Table S5. Group 1, withers heights. Table S6. Group 2, withers heights. Table S7. Group 3, withers heights. Table S8. Group 1, weights and measurements of the heart and spleen. Table S9. Group 2, weights and measurements of the heart and spleen. Table S10: Group 3, weights and measurements of the heart and spleen.
