# Supplementary figures and images for: Assessment of Minimum Stable Areas for Young Ostriches According to Animal Welfare Legislation
Source: Animals (Basel). 2025 Feb 18;15(4):582. doi: 10.3390/ani15040582 (PMC11851422; doi:10.3390/ani15040582)

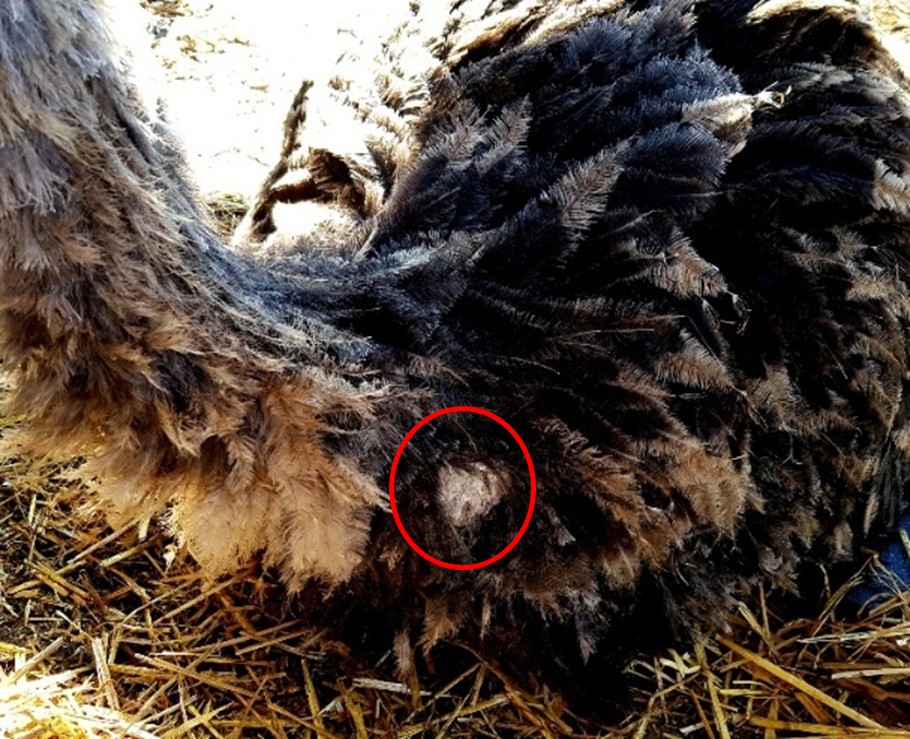

Supplement: Supplementary file 1 [file animals-15-00582-s001.zip › Figure S10_Group 1, bird with loss of feathers.jpg]

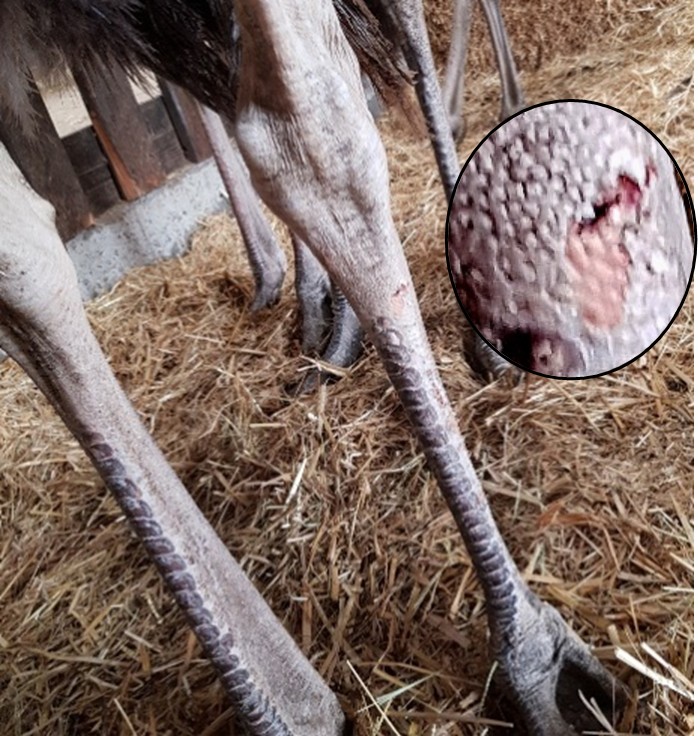

Supplement: Supplementary file 1 [file animals-15-00582-s001.zip › Figure S11_Group 3, bird with injury.jpg]

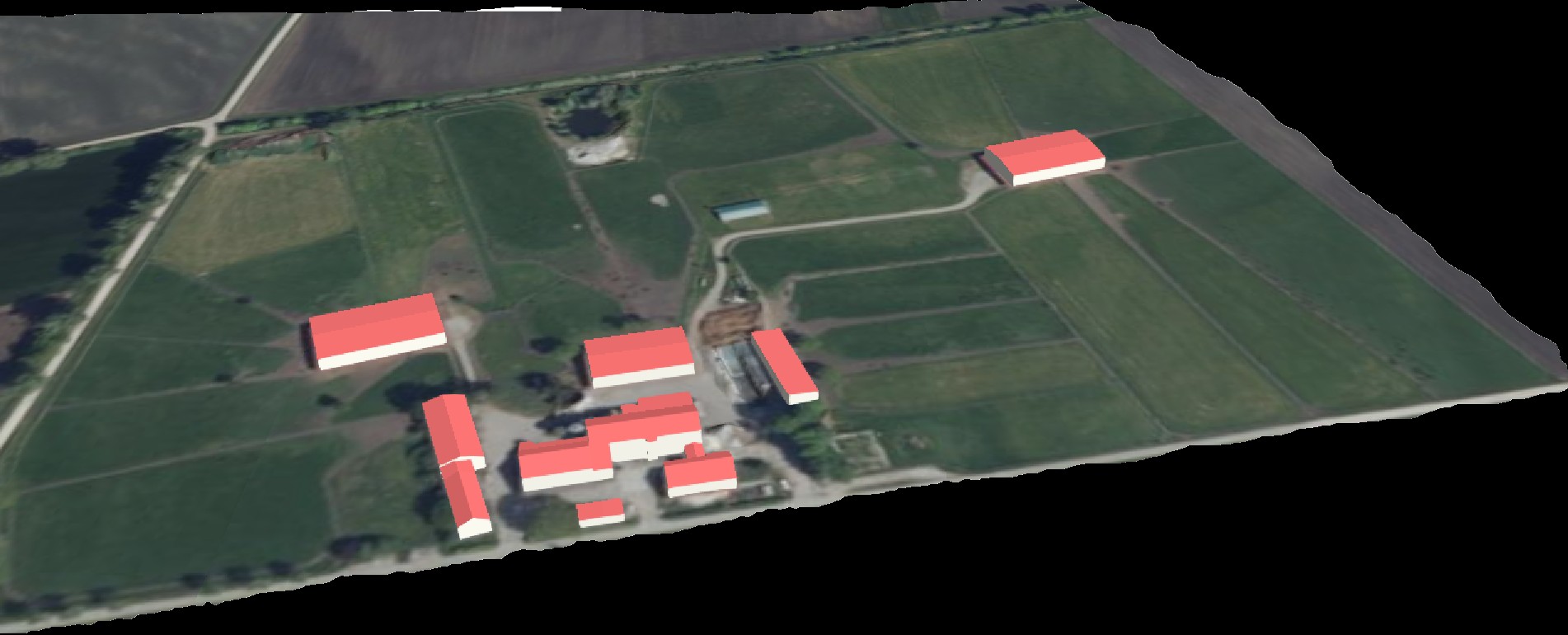

Supplement: Supplementary file 1 [file animals-15-00582-s001.zip › Figure S1_Aerial view of the ostrich farm.jpg]

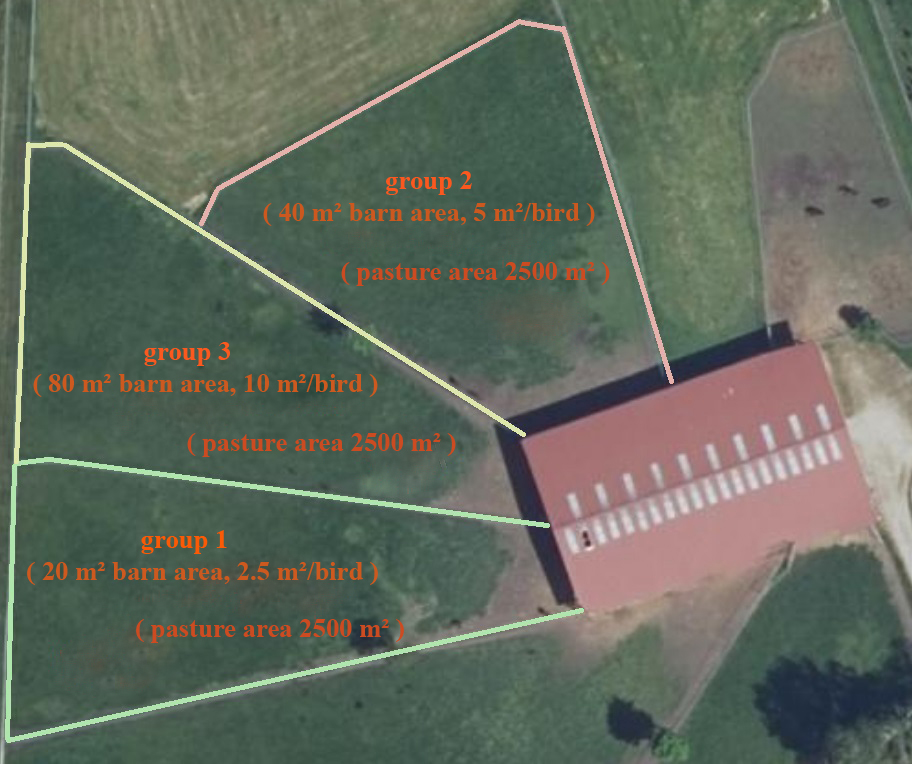

Supplement: Supplementary file 1 [file animals-15-00582-s001.zip › Figure S2_Aerial view of the pasture area.jpg]

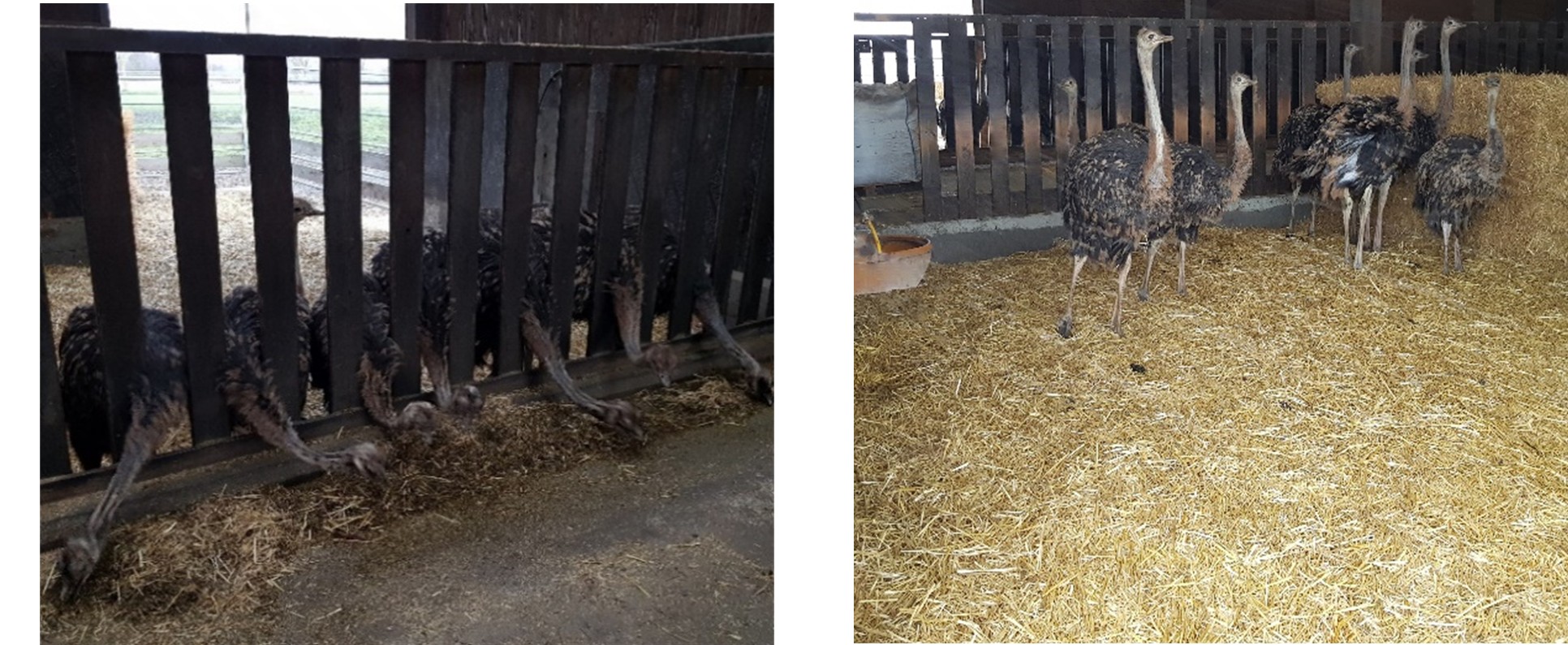

Supplement: Supplementary file 1 [file animals-15-00582-s001.zip › Figure S3_Barn and animals of Group 1 at the beginning of the study.jpg]

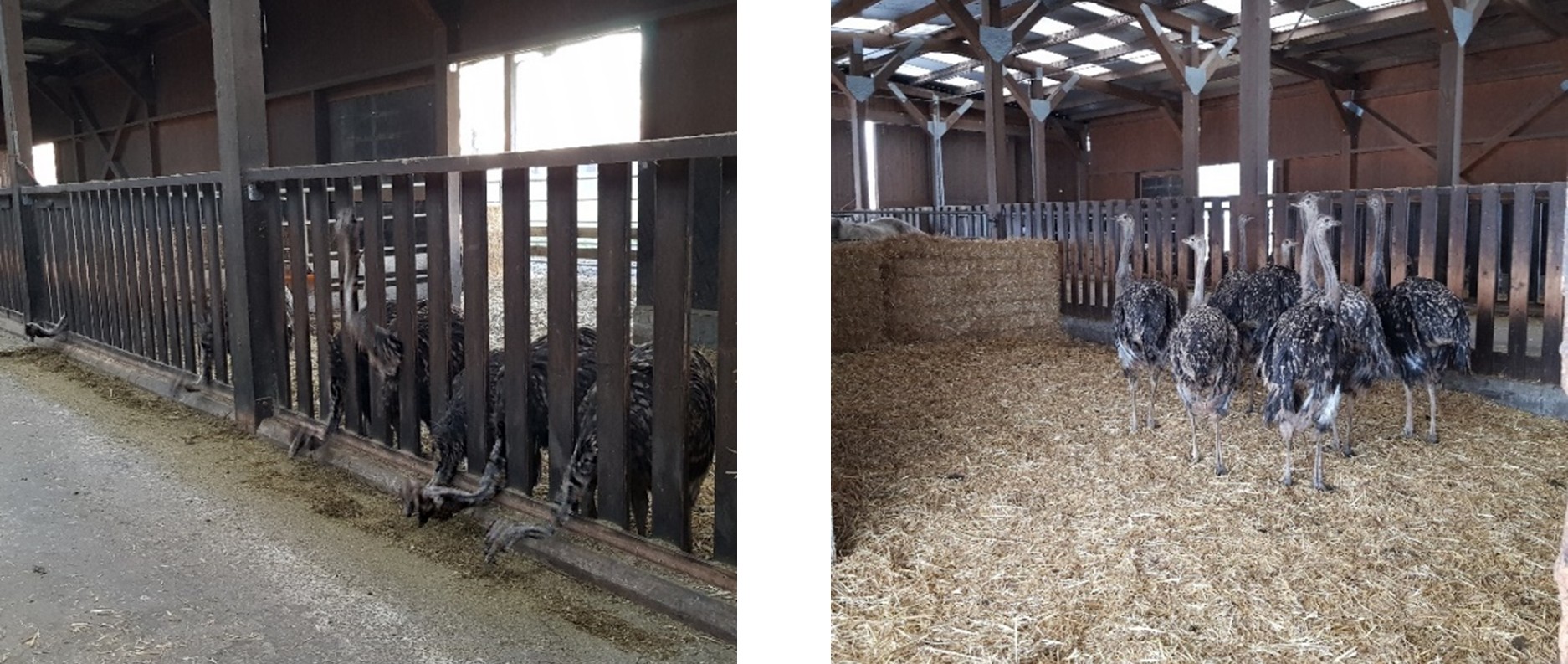

Supplement: Supplementary file 1 [file animals-15-00582-s001.zip › Figure S4_Barn and animals of Group 2 at the beginning of the study.jpg]

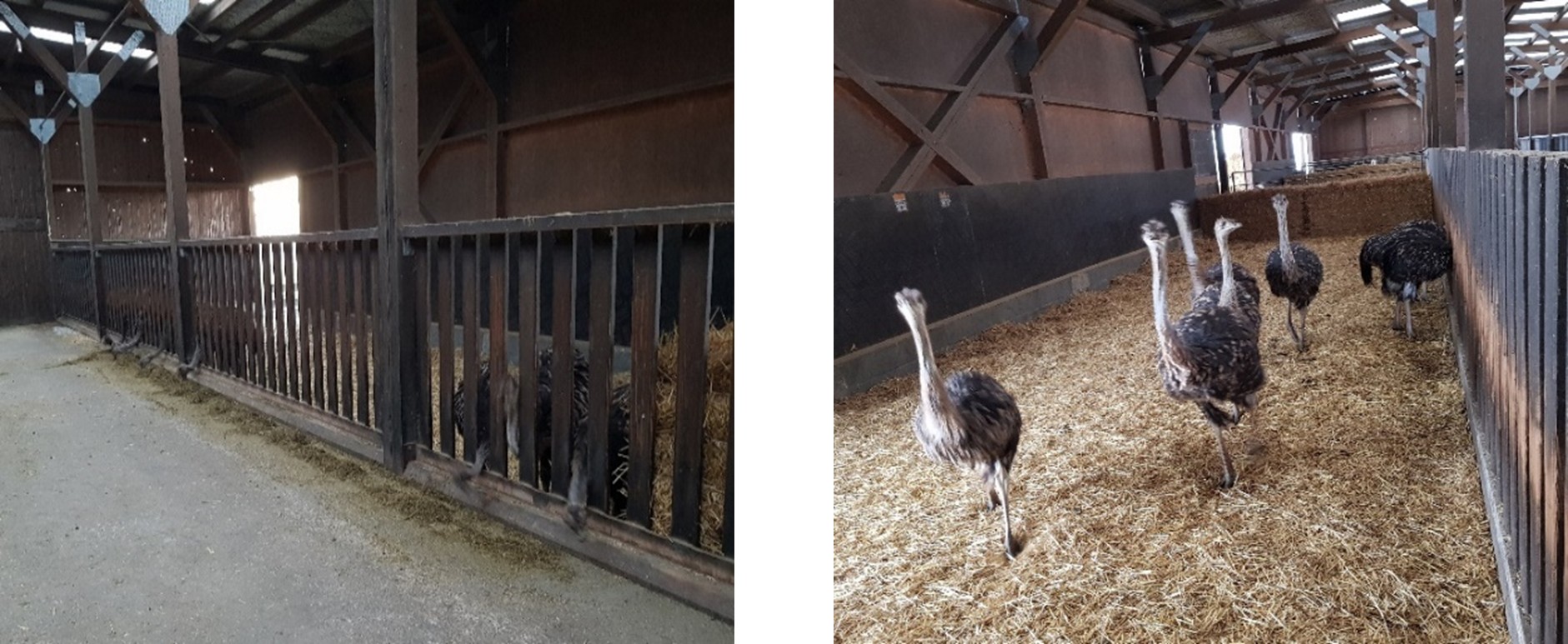

Supplement: Supplementary file 1 [file animals-15-00582-s001.zip › Figure S5_Barn and animals of Group 3 at the beginning of the study.jpg]

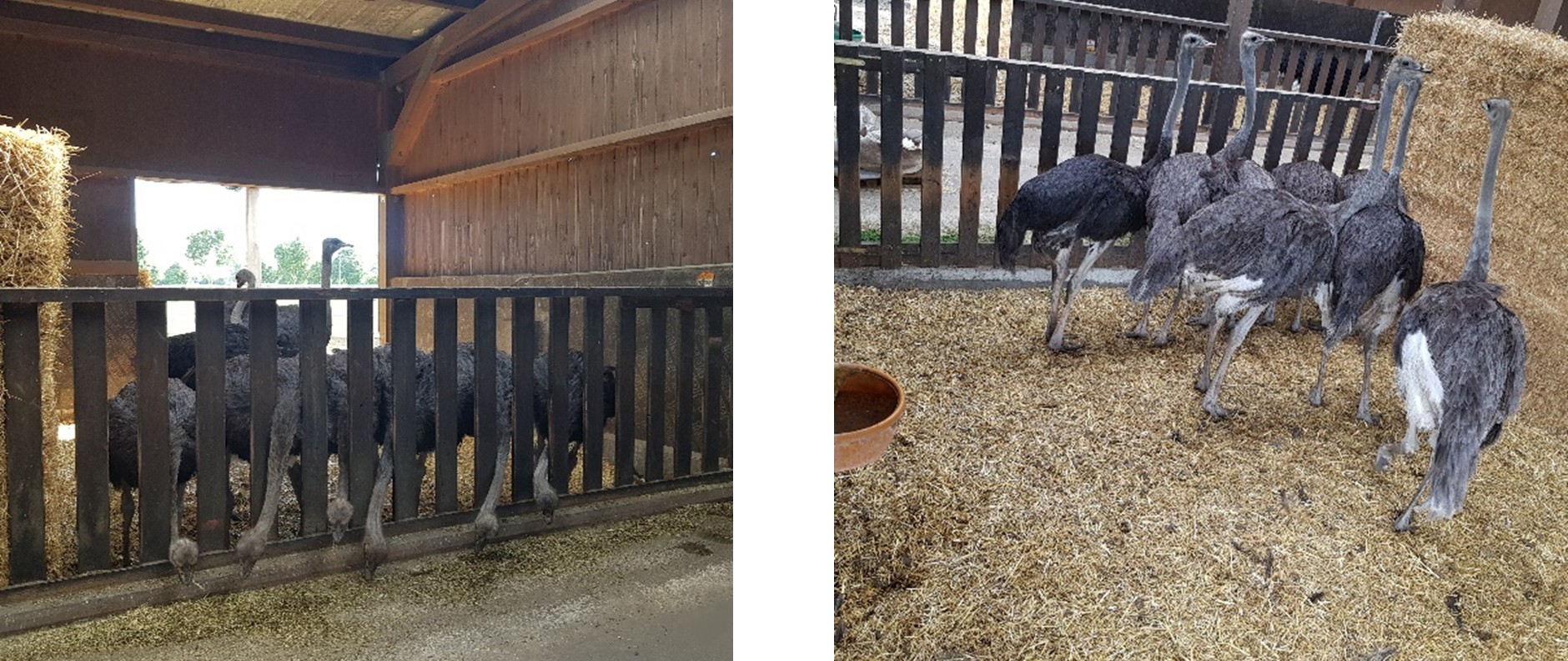

Supplement: Supplementary file 1 [file animals-15-00582-s001.zip › Figure S6_Barn and animals of Group 1 at the end of the study.jpg]

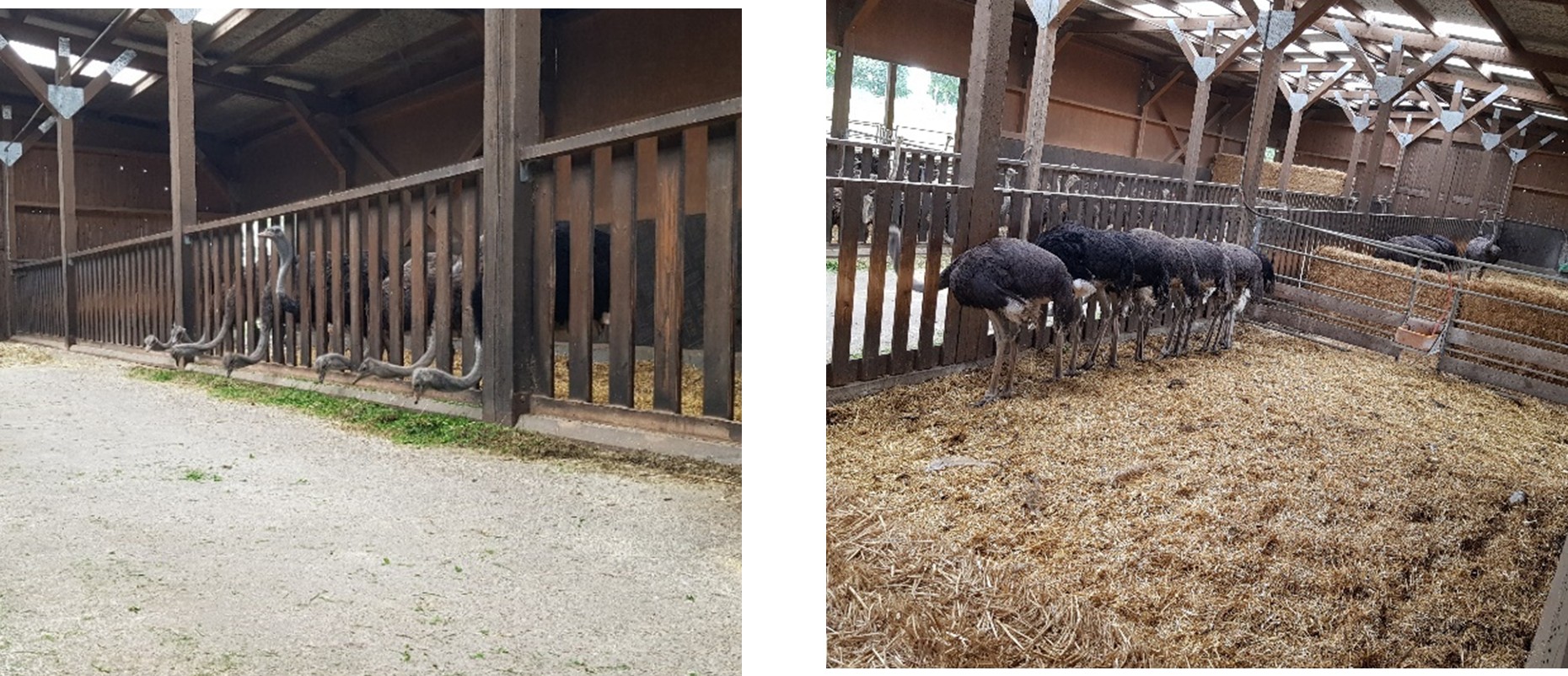

Supplement: Supplementary file 1 [file animals-15-00582-s001.zip › Figure S7_Barn and animals of Group 2 at the end of the study.jpg]

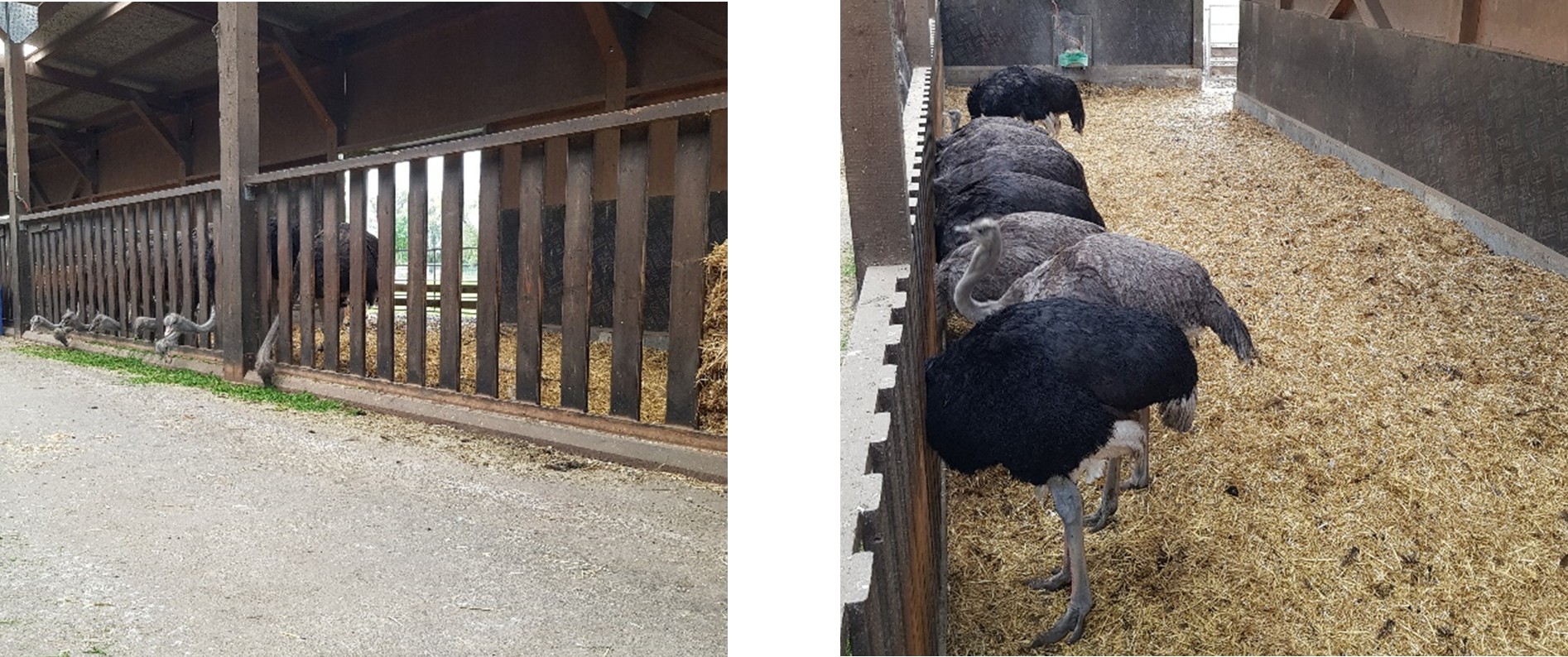

Supplement: Supplementary file 1 [file animals-15-00582-s001.zip › Figure S8_Barn and animals of Group 3 at the end of the study.jpg]

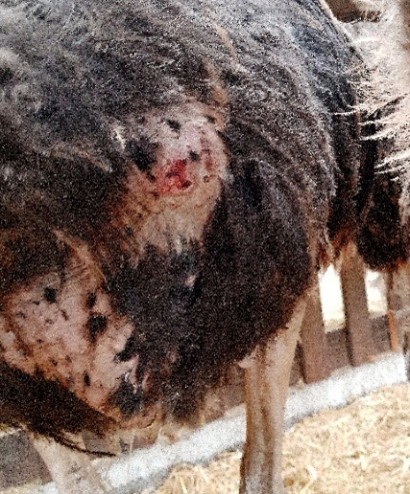

Supplement: Supplementary file 1 [file animals-15-00582-s001.zip › Figure S9_. Group 1, bird with injury.jpg]

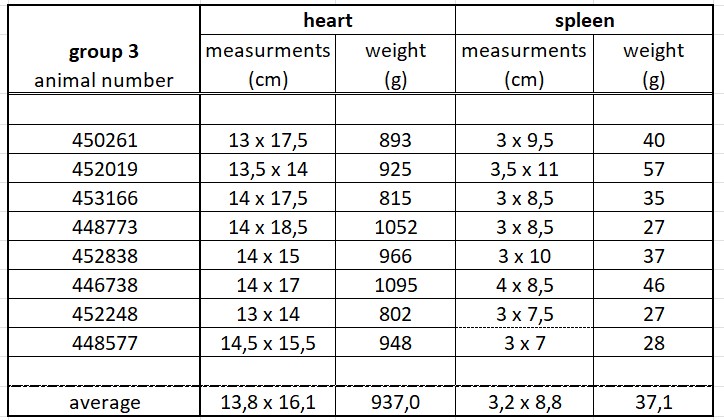

Supplement: Supplementary file 1 [file animals-15-00582-s001.zip › Table S10_Group 3, weights and measurements of the heart and spleen..jpg]

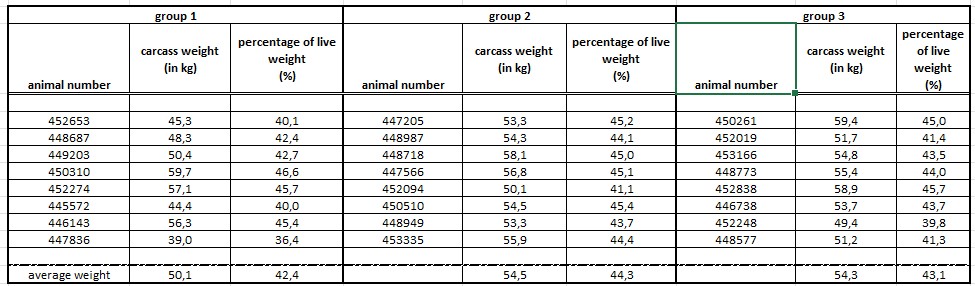

Supplement: Supplementary file 1 [file animals-15-00582-s001.zip › Table S1_Carcass weights.jpg]

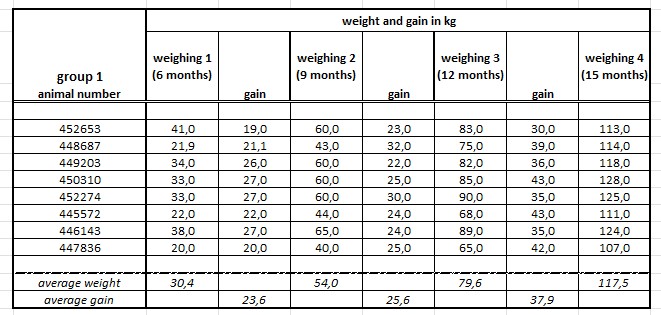

Supplement: Supplementary file 1 [file animals-15-00582-s001.zip › Table S2_Group 1, weights and gain.jpg]

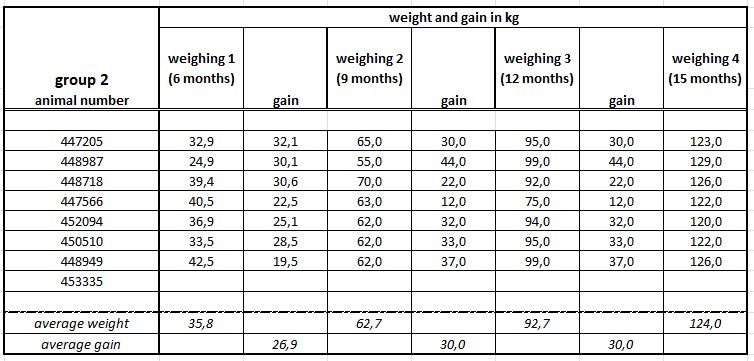

Supplement: Supplementary file 1 [file animals-15-00582-s001.zip › Table S3_Group 2, weights and gain.jpg]

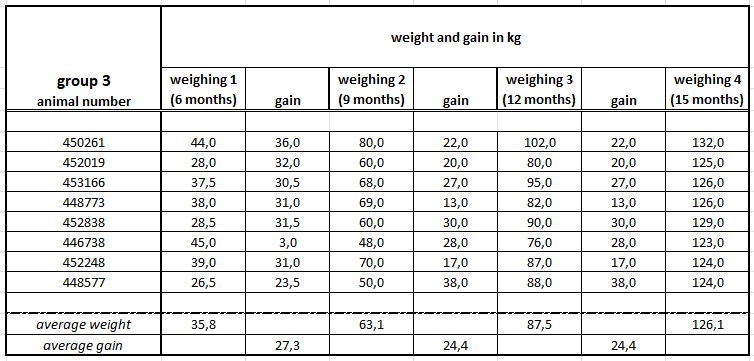

Supplement: Supplementary file 1 [file animals-15-00582-s001.zip › Table S4_Group 3, weights and gain.jpg]

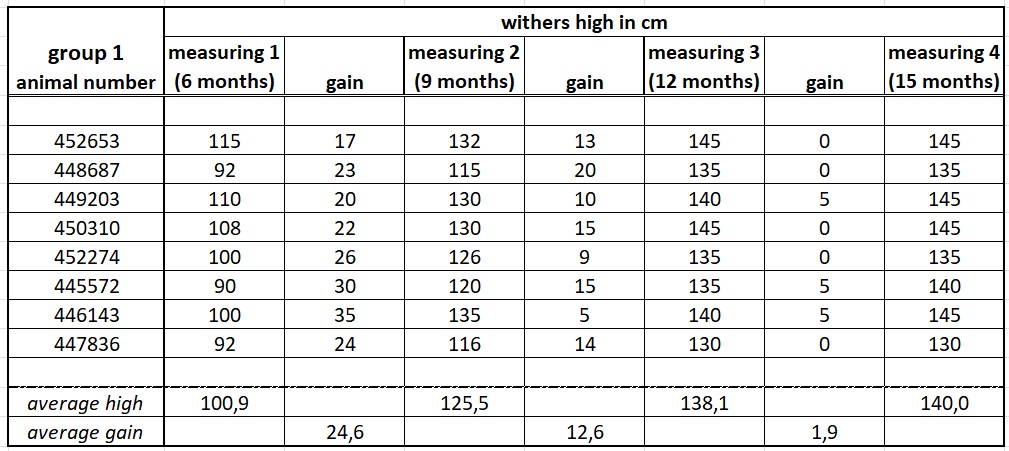

Supplement: Supplementary file 1 [file animals-15-00582-s001.zip › Table S5_Group 1, withers heights.jpg]

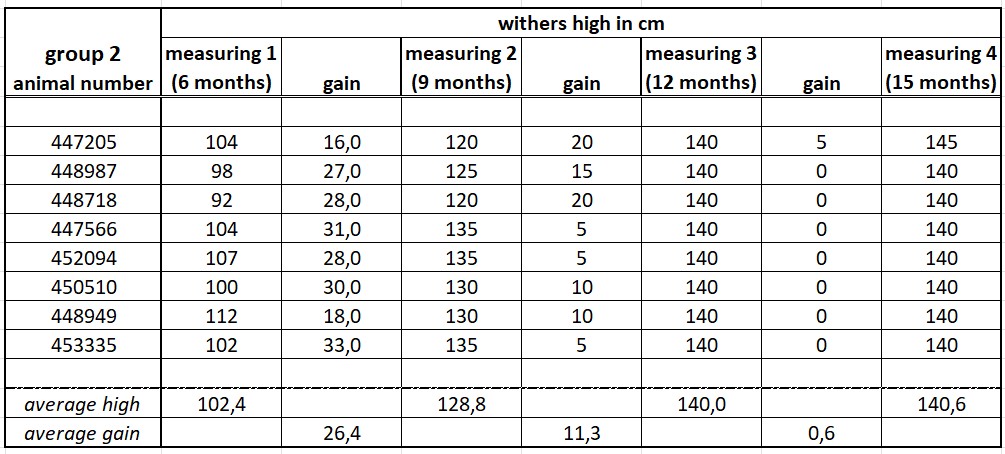

Supplement: Supplementary file 1 [file animals-15-00582-s001.zip › Table S6_Group 2, withers heights.jpg]

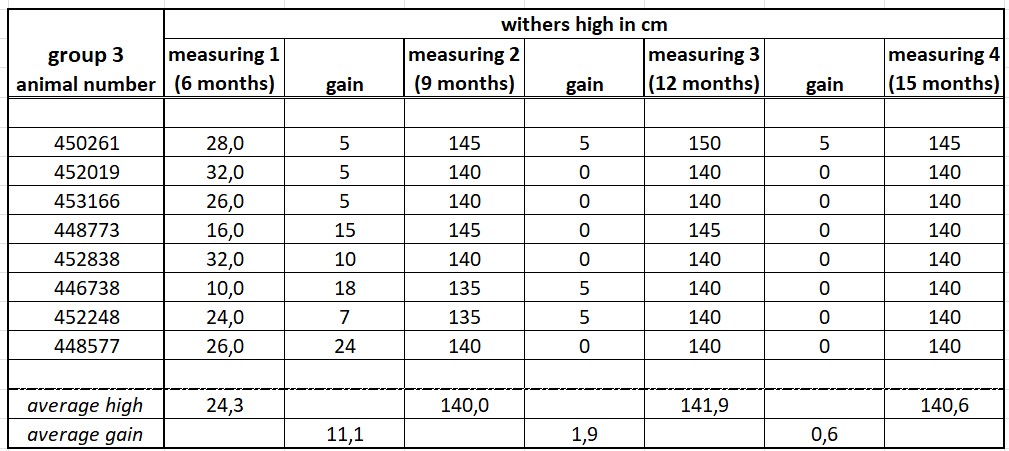

Supplement: Supplementary file 1 [file animals-15-00582-s001.zip › Table S7_Group 3, withers heights.jpg]

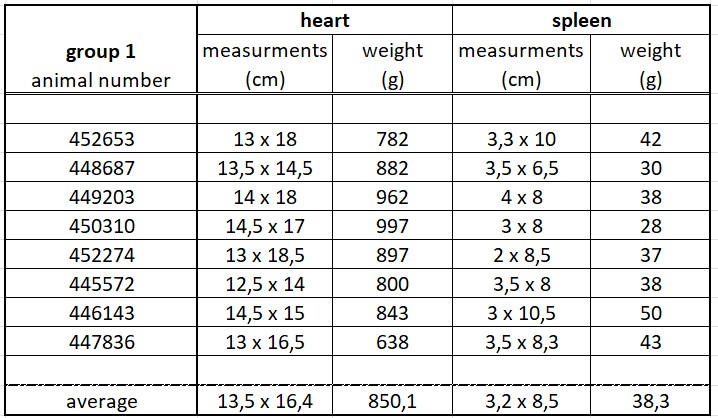

Supplement: Supplementary file 1 [file animals-15-00582-s001.zip › Table S8_Group 1, weights and measurements of the heart and spleen.jpg]

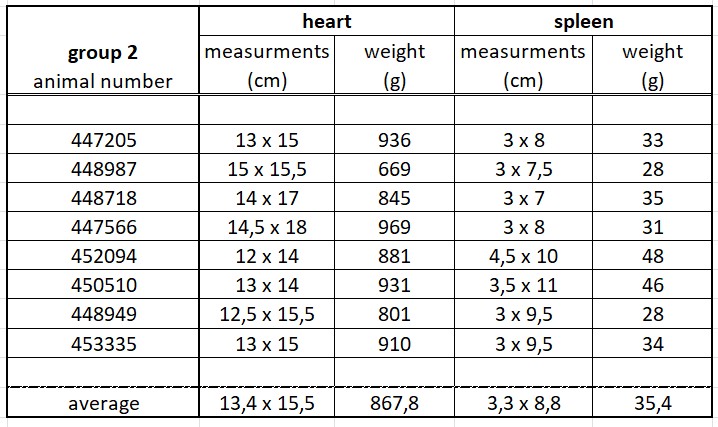

Supplement: Supplementary file 1 [file animals-15-00582-s001.zip › Table S9_Group 2, weights and measurements of the heart and spleen.jpg]
